# Public knowledge and attitudes towards antibiotic use across England – pre- and post-pandemic

**DOI:** 10.1186/s12889-025-25233-3

**Published:** 2025-11-21

**Authors:** Ellie Gilham, Brieze Read, Sarah Tonkin-Crine, Monsey McLeod, Diane Ashiru-Ordedope, Kate Duxbury, Colin S. Brown, Donna M. Lecky

**Affiliations:** 1 Antimicrobial Resistance and Healthcare-Associated Infection Division, United Kingdom Health Security Agency, London, UK; 2 Nuffield Department of Primary Care Health Sciences, University of Oxford, Oxford, UK; 3 National Institute for Health Research (NIHR) Health Protection Research Unit (HPRU) in Healthcare Associated Infections and Antimicrobial Resistance, University of Oxford, Oxford, UK; 4 Antimicrobial Resistance Programme, Medical Directorate, NHS England, London, UK; 5 Department of Medicine, Health Protection Research Unit in Healthcare Associated infections and Antimicrobial Resistance, Imperial College London, London, UK; 6 Ipsos UK Ltd, London, UK

**Keywords:** Antibiotic use, Public knowledge, Public attitudes, Antimicrobial resistance

## Abstract

**Background:**

Antibiotic misuse is a major preventable driving factor for antimicrobial resistance (AMR). Most antibiotics are prescribed in primary care where demand for consultations for common self-limiting infections is greatest, meaning public knowledge may influence antibiotic prescribing. This study aims to explore how public knowledge of and attitudes towards antibiotics have changed over time.

**Methods:**

Ipsos conducted interviews as part of routine surveys across England in 2020, 2021, 2022 and 2024. Random and quota sampling were used to ensure a representative sample. Questionnaire responses were weighted to ensure the results are broadly representative of the population. Pearson’s Chi-squared test was used to test for differences in proportions across levels of categorical variables and between responses across the four years.

**Results:**

Responses were obtained from 2,022 (pre-pandemic); 1,676 (pandemic-Y1); 1,663 (pandemic-Y2) and 3,024 (post-pandemic) respondents.

The proportion of respondents who felt they had personal responsibility to tackle AMR increased from 57% pre-pandemic to 62% in pandemic-Y1 (*p* < 0.05), reducing to 46% post-pandemic. The proportion of respondents correctly answering the statement *antibiotics will always speed up my recovery from an infection* increased from 58% pre-pandemic to 65% in pandemic-Y1 and Y2 (*p* < 0.05), reducing to 56% post-pandemic. Knowledge regarding the use of antibiotics to treat ear, urine infections and COVID-19 was lowest post-pandemic.

Trust in healthcare professionals (HCPs) regarding whether antibiotics are needed peaked during (range: 77% to 91%) and declined post-pandemic (range: 72% to 86%). The proportion of respondents who reported they would be pleased if their GP did not prescribe antibiotics was highest pre-pandemic (84%), decreasing to 65% post-pandemic. The proportion of respondents who were likely to request antibiotics from their GP declined from pre-pandemic (21%) to pandemic-Y1 (19%) but increased post-pandemic (25%). Demographic variations were observed across nearly all questions.

**Conclusions:**

This paper highlights some concerning trends. Knowledge regarding AMR and the specific infections that antibiotics can treat has reverted to pre-pandemic levels, while levels of uncertainty about AMR and antibiotic use have increased. Although high, trust in HCPs has declined. Therefore, future interventions may wish to support HCPs to build trust with their patients and consider how care pathways can promote this.

**Supplementary Information:**

The online version contains supplementary material available at 10.1186/s12889-025-25233-3.

## Introduction

Antimicrobial resistance (AMR) is a leading cause of death worldwide, with nearly five million deaths estimated to be associated with AMR in 2019 [1]. Overuse and inappropriate use of antimicrobial medicines are preventable drivers of AMR [2]. A key part of the World Health Organisation’s strategy for addressing AMR is raising awareness of AMR through the co-ordination of national action plans (NAPs) in 148 countries, and increasing levels of public engagement [3]. The UK’s 2024–2029 NAP includes goals to reduce total antibiotic use by 5% and increase public knowledge of AMR [4].

The greatest proportion of antimicrobial prescribing occurs in primary care where a high proportion of infection consultations are for common self-limiting infections [5]; a setting where perceived patient demand, patient expectations for antibiotics and perceived difficulties in maintaining patient relationships are identified, amongst others, as drivers of inappropriate prescribing [6–10]. Therefore, reducing patient expectations for antibiotics through improvements in public knowledge and attitudes towards AMR and antibiotic use may help to mitigate inappropriate prescribing in this sector.

**Fig. 1:**
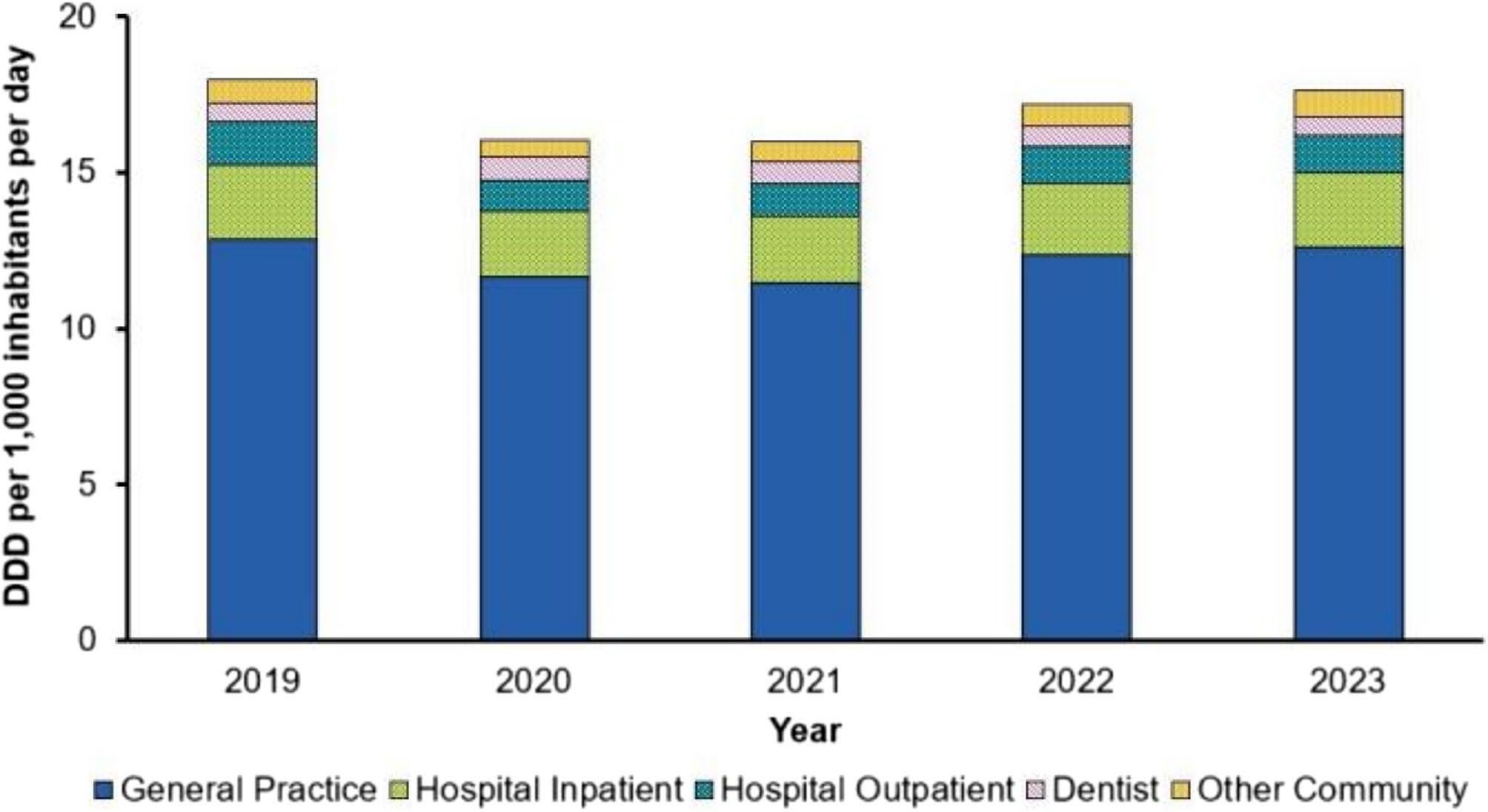
Total antibiotic consumption within general practice, hospital inpatient, hospital outpatient, dentist and other community settings, expressed as defined daily doses (DDD) per 1,000 inhabitants per day (DID) in England from 2019 to 2023. Permission was gained to use this figure which was originally published in the 2023-24 ESPAUR report [6]

Several national campaigns aimed at improving public awareness and knowledge of AMR and appropriate antibiotic use were run prior to the COVID-19 pandemic [11, 12]. However, the pandemic placed a hiatus on many national and global initiatives for antimicrobial stewardship (AMS) [13]. During the COVID-19 pandemic, antimicrobial prescribing in the UK initially spiked, and then dropped to below the previous year’s prescribing rates. However, they immediately began to rise following lifting of the third lockdown restrictions (July 2021) and continued to increase until in line with the expected trend [13], returning to pre-pandemic levels in 2023 [5] (Fig. 1.). During and in the time following the COVID-19 pandemic, patient education on prudent antibiotic use has been reliant on healthcare professionals (HCPs) providing resources to patients during consultations, such as use of the TARGET antibiotics patient information leaflets during primary care consultations [14], which are hosted on the RCPG website (https://elearning.rcgp.org.uk/course/view.php?id=553).

The present study aims to explore how public knowledge of, and attitudes towards, AMR and antibiotic use has changed during and following the COVID-19 pandemic.

## Methods

The market research company Ipsos MORI conducted interviews as part of routine surveys across England in 2020 (findings published by McNulty et al. 2022 [15]), 2021 and 2022 and across the UK in 2024, with only data from England reported in the present study. The questionnaires used were developed over time using a knowledge-attitude-behaviour framework with input from behavioural scientists [16]. The questionnaires aim to determine gaps within the public’s knowledge of AMR and to better understand what may be motivating their health-seeking behaviours. Details on the data collection methodology for the baseline [15] and subsequent surveys are outlined in Table 1. Results are reported from questions pertaining to antibiotic use in the preceding 12-months and their current knowledge, therefore the data collected from January to February 2020 was used as the pre-pandemic baseline. Partially completed interviews were excluded.

**Table 1:** Data collection methodology across the four years the surveys were conducted

Year	Data collection period	Data collection method
Pre-pandemic/baseline (2019–2020)	24th January to 2nd February 2020	Face-to-face in the interviewees’ own home via computer-aided personal interviews (CAPI)
Pandemic-Y1 (2020–2021)	26th February to 2nd March 2021	Computer-aided telephone interviews (CATI)
Pandemic-Y2 (2021–2022)	26th February to 2nd March 2022	Computer-aided telephone interviews (CATI)
Post-pandemic (2023–2024)	15th March to 27th March 2024	Online questionnaire (Computer Assisted Web Interviewing (CAWI))

Representativeness of the sample was ensured in 2020 by two-stage random sampling [17], with a random location sampling method used to select sample points in each region and interviewer quotas then set for gender, age, working status and region. In 2021 and 2022 representativeness was ensured by random digit dialling, and publicly available targeted data (see Supplementary Material 1.), and in 2024 by the use of quotas set on age, gender, region and working status. To ensure the results are broadly representative of the adult population, CAPI/CATI/CAWI uses a random iterative method weighting system to correct for known selection biases. It weights survey data to the latest set of census data or mid-year estimates and national readership survey profiles for age, social grade, region and working status, within gender and additional profiles on tenure and ethnicity. To further increase the sample size in order to allow for subgroup analysis, ethnic minorities were grouped into a non-white category. Pearson’s Chi-squared test, corrected for survey design, was used to test for differences in proportions across levels of categorical variables and between years, up to 2022. Statistical significance testing has not been conducted on the differences between the 2022 and 2024 surveys due to the changes in survey methodology, as well as wording changes to some of the survey questions.

For knowledge and attitude questions in the pre-pandemic (2020), pandemic-Y1 (2021) and pandemic-Y2 (2022), questionnaires respondents were presented with a series of statements and asked to what extent they believed the statement was ‘*definitely true*’ (4), ‘*true*’ (3), ‘*false*’ (2), or ‘*definitely false*’ (1). The ‘definitely true’ and ‘true’ responses were collated and reported as ‘true’, the same process was used for ‘definitely false’ and ‘false’ responses. In the 2024 questionnaire, only the ‘true’ or ‘false options were provided. These responses were then recoded as correct or incorrect. An additional ‘Unsure’ option was also provided; this was interpreted in the results and discussion section as uncertainty regarding the statement rather than as an incorrect response or negative attitude.

## Results

Responses were obtained from 2,022 (pre-pandemic); 1,676 (pandemic-Y1); 1,663 (pandemic-Y2) and 3,024 (post-pandemic) respondents. Respondent demographics were similar across all four time-points as shown in Supplementary Material 2.

### Antibiotic use

Approximately one-quarter reported taking antibiotics within the previous 12-months (pre-pandemic 29%; pandemic-Y1 25%; pandemic-Y2 28%; post-pandemic 32%). Pre and during the pandemic there were no notable differences across demographics. Post-pandemic reported antibiotic use was higher in younger adults aged 16 to 34-years (44%), respondents from ethnic minority backgrounds (42%), those with a disability (44%), and in the lowest Index of Multiple Deprivation (IMD) quintile (37%).

### Attitudes towards antimicrobial stewardship

**Fig. 2:**
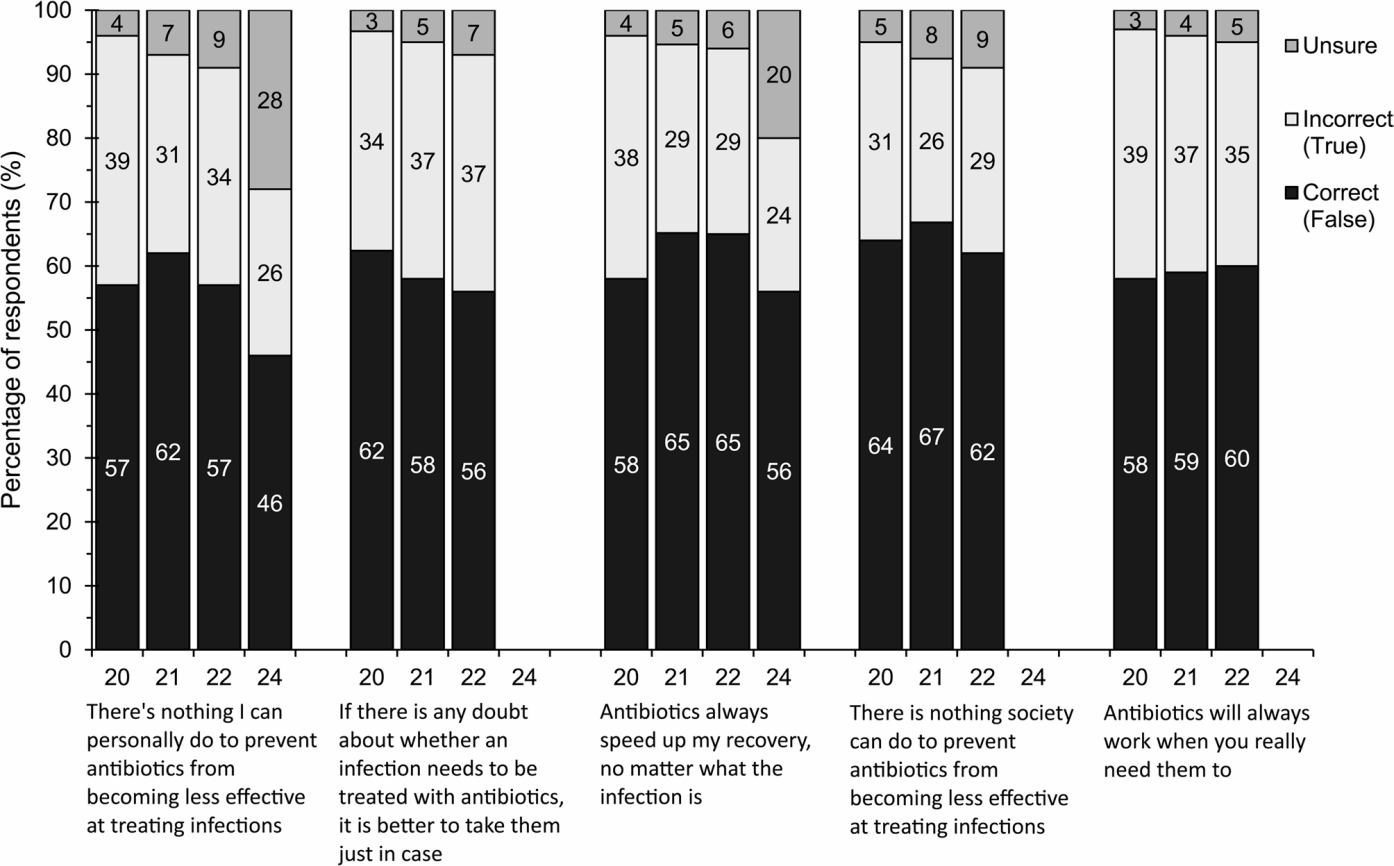
Percentage of respondents answering true or false to five knowledge statements in pre-pandemic (2020), pandemic Y1 (2021), pandemic Y2 (2022) and post-pandemic (2024). Statements *If there is any doubt about whether an infection needs to be treated with antibiotics, it is better to take them just in case*,*There is nothing society can do to prevent antibiotics from becoming less effective at treating infections*, and *Antibiotics will always work when you really need them to* were not included in the post-pandemic (2024) questionnaire

Figure 2 outlines respondents’ attitudes towards AMS and antibiotic use. The proportion of respondents who felt the statement ‘*there is nothing society can do to prevent antibiotics from becoming less effective at treating infections*’ is false increased during pandemic-Y1 then reduced back to baseline in pandemic-Y2 (pre-pandemic, 64%; pandemic-Y1, 67%, *p* < 0.05; pandemic-Y2 62%, *p* < 0.05.). This question was not asked in the post-pandemic questionnaire.

The proportion of respondents who felt the statement ‘*there’s nothing I can personally do to prevent antibiotics from becoming less effective at treating infections’* is false was highest in pandemic-Y1 (62%), increasing by 5% from pre-pandemic levels (*p* < 0.05). This then reduced to the pre-pandemic baseline in pandemic-Y2 (57%) and declined further post-pandemic (46%). A concurrent increase in those stating they were unsure whether they agreed with the statement (pre-pandemic, 4% vs. post-pandemic, 28%) was seen in line with the decrease in positive attitudes observed in 2024 (Supplementary Material 3.).

Demographical differences for these statements followed similar trends with a higher proportion of positive responses seen in social grades ABC1 (those in higher managerial, administrative or professional, intermediate managerial, supervisory/clerical, junior managerial, administrative or professional roles) compared to those from social grades C2DE (skilled manual workers, semi and unskilled manual workers, state pensioners or widows (no other earner), casual or lowest grade workers), those educated to a degree level compared to those educated to less than degree level, and white respondents compared to respondents from ethnic minority backgrounds. Attitudes also varied with age with the proportion of respondents answering positively highest in the 25 to 54 age groups and lowest in the 16 to 24 and over 55-years age groups for the first three years. However, in post-pandemic it was highest in the 55 to 64 and 35 to 44 and lowest in the 16 to 24 and 45 to 54 age groups (Supplementary Material 4 to 7.).

### Knowledge of AMR and antibiotic usage

Knowledge regarding the use of antibiotics to speed up recovery improved during the pandemic. The proportion of individuals correctly responding to the statement ‘*antibiotics always speed up your recovery*,* no matter what the infection is’* (correct response = false) increased by 7% from pre-pandemic (58%) to pandemic-Y1 (65%, *p* <.05). This was maintained in pandemic-Y2, and then declined post-pandemic to pre-pandemic levels (56%). The reduction seen in the proportion of respondents incorrectly answering the above statement post-pandemic corresponded with a large increase in the proportion of respondents stating they ‘*don’t know’*. The proportion of respondents disagreeing with the statement was highest in those from social grades ABC1 compared to C2DE, those educated to degree-level compared to those educated to less than degree level, white respondents compared to respondents from ethnic minority backgrounds, and in older adults.

Knowledge of AMR increased from pre-pandemic to pandemic-Y2 with the proportion of respondents correctly responding to the statement ‘*antibiotics will always work when you really need them’* increasing over time (pre-pandemic, 58%, pandemic-Y1, 59%; pandemic-Y2, 60%, *p* < 0.05). This question was not asked in the post-pandemic questionnaire. Following similar trends to those above, knowledge was higher in those from social grades ABC1 compared to C2DE, those educated to degree-level compared to those educated to less than degree level and in older adults. Differences with ethnicity were only seen pre-pandemic. Conversely, the proportion of respondents correctly responding to the statement ‘*if there is any doubt about whether an infection needs to be treated with antibiotics*,* it is better to take them just in case’* declined by 6% from pre-pandemic (62%) to pandemic-Y2 (56%, *p* < 0.05). The reduction in the proportion of correct responses in pandemic-Y2 corresponded to an increase in the proportion of respondents stating that they don’t know (pre-pandemic, 3%, pandemic-Y2, 7%). Respondents from social grades C2DE were more likely to agree with the statement that those from grades ABC1. This question was not asked in the post-pandemic questionnaire.

### Knowledge on use of antibiotics to treat specific infections

**Fig. 3:**
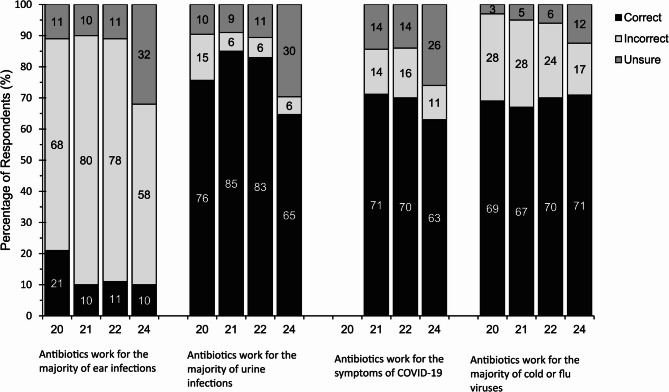
Percentage of respondents correctly answering whether antibiotics are effective at treating ear infections, urinary tract infections, COVID-19, and colds/flu in pre-pandemic (2020), pandemic-Y1 (2021), pandemic-Y2 (2022) and post-pandemic (2024) In 2022, a change in question wording occurred with the statement *Antibiotics do not treat COVID-19*. This changed from *Antibiotics work for the symptoms of COVID-19* to *Antibiotics do not treat COVID-19*. In 2024, a change in question wording occurred with statements *Antibiotics do not treat COVID-19* and *Antibiotics work for the majority of cold or flu viruses* to the following: *Antibiotics work for the symptoms of COVID-19* and *Antibiotics are effective against colds*

Knowledge on use of antibiotics to treat specific infections is shown in Fig. 3. The proportion of respondents who correctly identified that antibiotics work for the majority of urine infections, improved during the pandemic (pre-pandemic, 76%; pandemic-Y1, 85%, *p* < 0.05; pandemic-Y2 83%). This declined post pandemic with 65% of respondents correctly identifying that antibiotics can be used to treat urine infections. Knowledge on the use of antibiotics to treat urine infections tended to be higher among those who have taken an antibiotic in the last 12-months compared to those who hadn’t (pre-pandemic, 79% vs. 74%; pandemic-Y1, 88% vs. 84% pandemic-Y2, 88% vs. 81%, *p* < 0.05; post-pandemic 75% vs. 60%, *p* < 0.05).

Knowledge that antibiotics do not work for the majority of ear infections was highest pre-pandemic with 21% of respondents answering this question correctly. This decreased significantly in pandemic-Y1 (10%, *p* < 0.05) and has since remained stable (pandemic-Y2, 11%; post-pandemic, 10%). Similar to other question responses, the decrease in correct knowledge post-pandemic corresponded with an increase in those stating that they were unsure whether the statement was true or false, rather than an increase in an incorrect answer. A higher proportion of respondents who had taken antibiotics in the previous 12-months thought antibiotics were effective at treating ear infections compared to respondents who had not taken antibiotics in the previous 12-months (pre-pandemic, 69% vs. 68%; pandemic-Y1, 81% vs. 79% pandemic-Y2, 83% vs. 75%, *p* < 0.05; post-pandemic 66% vs. 54%, *p* < 0.05).

Knowledge that antibiotics don’t work for the majority of cold or flu infections remained consistent across the pandemic (pre-pandemic, 69%; pandemic-Y1, 67%; pandemic-Y2, 70%). Post-pandemic, 71% of respondents correctly identified that antibiotics are not effective against colds. In pandemic-Y1 and post-pandemic those who had taken antibiotics in the previous 12 months had poorer knowledge regarding the effectiveness of antibiotics at treating colds/flu (post-pandemic 63% vs. 75%, *p* < 0.05). During pandemic years this was comparable with those who knew that antibiotics are not effective against the symptoms of COVID-19 (pandemic-Y1, 71%; pandemic-Y2, 70%); however correct knowledge on antibiotic effectiveness against COVID-19 decreased to 63% post-pandemic. Furthermore, knowledge regarding the effectiveness of antibiotics at treating COVID-19 was poorer in those who had taken antibiotics in the previous 12 months post-pandemic (58% vs. 66%, *p* < 0.05).

Knowledge regarding the use of antibiotics for the treatment of colds and flu was significantly higher among females (Supplementary Material 8.), older age groups and white respondents and for urine infections, knowledge was significantly higher among females. Demographic differences in knowledge regarding the use of antibiotics for the treatment of ear infections and COVID-19 fluctuated between years (Supplementary Material 4 to 7.).

### Attitudes towards prescribing

**Fig. 4:**
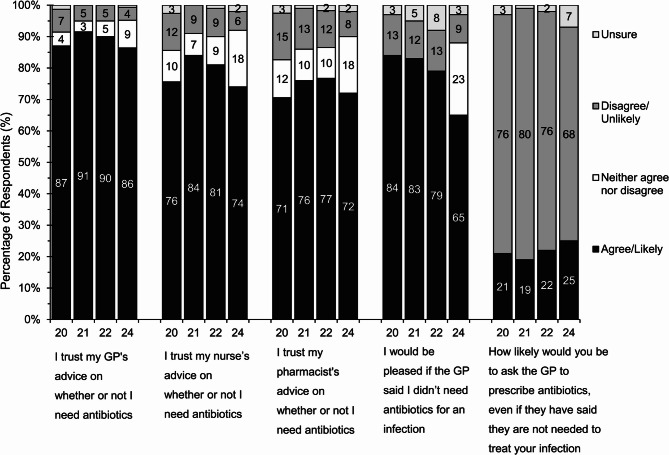
Responses to statements reflecting attitudes towards antibiotic prescribing in pre-pandemic (2020), pandemic-Y1 (2021), pandemic-Y2 (2022) and post-pandemic (2024). For the statement *I would be pleased if the GP said I didn’t need antibiotics for an infection*, from pre-pandemic to pandemic-Y2 the questions responses were true/false. This changed to agree/disagree post-pandemic with a neither agree nor disagree response option also added

Trust in HCPs’ advice on the need for antibiotics increased significantly during pandemic-Y1 (general practitioner (GP) 91%; nurse 84%; pharmacist 76%) compared to pre-pandemic levels (GP 87%, *p* < 0.05; nurse 76%, *p* < 0.05; pharmacist 71%, *p* < 0.05) (Fig. 4.). Trust remained high in pandemic-Y2 (GP 90%, *p* < 0.05; nurse 81%, *p* < 0.05; pharmacist 77%, *p* < 0.05) but has returned to baseline levels post-pandemic (GP 87%; nurse 75%; pharmacist 73%). Trust in GP, nurse and pharmacists’ advice tends to be higher in white respondents compared to those from ethnic minority backgrounds across all four-years (Supplementary Material 6.).

Four in five respondents (pre-pandemic, 84%; pandemic-Y1, 85%; pandemic-Y2, 80%) stated ‘*they would be pleased if their GP said they didn’t need antibiotics for an infection’*. Older age groups (45 to 54 and 55 to 64-year-olds) were more likely to agree with this statement than 18 to 24-year-olds (pre-pandemic, 89% and 88% vs., 80%; pandemic-Y1, 86% and 87% vs. 77% (*p* < 0.05); pandemic-Y2 83% and 83% vs. 80%, respectively). Post-pandemic, 65% of respondents agreed that they would *be pleased if their GP said they didn’t need antibiotics for an infection.* This was significantly higher in those aged over 45 compared to those aged 16 to 44-years (Supplementary Material 7.). Across all four years, those disagreeing with the statement (i.e., those who would not be pleased if told antibiotics were not required for their infection) was significantly higher in those who have taken an antibiotic in the last 12 months, compared to those who haven’t (pre-pandemic 17% vs. 11%, *p* < 0.05; pandemic-Y1 18% vs. 10%, *p* < 0.05; pandemic-Y2, 17% vs. 12%, *p* < 0.05; post-pandemic, 12% vs. 7%, *p* < 0.05).

Respondents were asked how likely they were to request antibiotics from their GP if they felt antibiotics were needed for their illness, but their GP advised that they are not. One in five said they were likely to request antibiotics from their GP (pre-pandemic, 21%; pandemic-Y1, 19%; pandemic-Y2, 21%) increasing to one in four post-pandemic (25%). Across all four years those who have taken antibiotics within the previous 12-months (pre-pandemic, 27% vs. 19%, *p* < 0.05; pandemic-Y1, 26% vs. 16%, *p* < 0.05; pandemic-Y2, 26% vs. 20%, *p* < 0.05; post-pandemic, 40% vs. 18%, *p* < 0.05) and respondents from ethnic minority backgrounds (pre-pandemic 37% vs. 18%, *p* < 0.05; pandemic-Y1 34% vs. 16%, *p* < 0.05; pandemic-Y2 35% vs. 19%, *p* < 0.05; post-pandemic 22% vs. 41% *p* < 0.05) were significantly more likely to request antibiotics from their GP than those who had not taken antibiotics in the previous 12 months. Post pandemic, those who trusted their GP’s advice on whether or not an antibiotic was needed were less likely to request antibiotics compared to those who did not trust their GPs advice (91% vs. 77%, *p* < 0.05).

## Discussion

This paper highlights the public’s current level of knowledge of, and attitudes towards AMR and antibiotic use and demonstrates how these have changed over the COVID-19 pandemic. The findings build on our current level of understanding and will allow the implementation of more targeted interventions to facilitate progress towards public engagement targets published within the 2024–2029 NAP [4].

Prior to the pandemic, the Keep Antibiotics Working campaign [18] ran during the winters of 2017 to 2019. It aimed to raise awareness that taking antibiotics when not needed will result in them becoming ineffective and encouraged the public to trust their HCPs’ advice [19]. Amidst the pandemic, there were concerns that the absence of such a public campaign, coupled with the barrage of pandemic-related health messages predominately focused on hand hygiene and other infection prevention behaviours [20], could deprioritise AMS [21, 22] and negatively impact public awareness of appropriate antibiotic consumption.

Nevertheless, knowledge regarding AMR increased during the pandemic. The proportion of those who thought *antibiotics will always work when they need them* reduced by 4% from pre- to pandemic-Y2. There was also a heightened public belief that they themselves (62%), and society (67%), have a role in combatting AMR. However, these beliefs declined slightly in the second year of the pandemic, possibly as an impact of messaging fatigue [23] as well as the extended nature of the pandemic and the negative impact this had on many individuals mental health [24]. Furthermore, the belief that they have a role in tackling AMR has since declined further post-pandemic. This reduction occurred concurrently with a large increase in respondents reporting they don’t know if they have a role in tackling AMR. Knowledge regarding the use of antibiotics to speed up recovery has also since returned to pre-pandemic levels again with a concurrent increase in those reporting uncertainty regarding this statement.

The exception was for knowledge relating to using antibiotics *just in case if there is any doubt that they are needed* with the proportion of people correctly answering this statement declining year-on-year during the pandemic. Reasons for this reduction are unclear, although the high levels of media coverage seen during the pandemic may have heightened public awareness of, and increased anxiety towards, the severe consequences that infectious diseases can have, as seen during the COVID-19 pandemic [25]. Fear of the consequences of an infection have been shown to result in patients requesting antibiotics from their HCP, especially within paediatric populations [26]. This was highlighted by the invasive group A *Streptococcus* outbreak in 2022 that resulted in high levels of prescribing for sore throat, which may have fuelled the attitude that antibiotics should be taken just in case [27].

With the exception of cold and flu, public knowledge of which infections can be effectively treated with antibiotics has declined post-pandemic. Prior to the pandemic, knowledge that antibiotics do not treat most ear infections [28] was low, which is consistent with previous findings [15], and has since declined sharply. Furthermore, previous antibiotic use was associated with lower knowledge of appropriate antibiotic use for ear infections. It is unclear if poorer knowledge in this area has been driven by inappropriate prescribing for ear infections during the pandemic as prescribing data is difficult to disaggregate in order to differentiate between ear infections and generic upper respiratory tract infections. However, it is worth noting that a small proportion (7%) of respondents reported having had an ear infection in the previous 12-months, meaning this finding may not be generalisable to the wider public. Colds and flu have been the primary focus of previous campaigns [11, 19] which may explain the higher levels of knowledge regarding the effectiveness of antibiotics at treating these infections and suggests that campaigns that aim to support knowledge for other infections, such as ear infections, may be beneficial.

Uncertainty regarding whether antibiotics can be used to treat urinary tract infections and COVID-19 has also increased by 21% and 12% respectively from the first year of the pandemic to post-pandemic. Interestingly, antibiotic use in the previous 12-months improved knowledge for urine infections and had no effect on knowledge for colds and flu nor COVID-19 prior to or during the pandemic. However, post-pandemic those who had been prescribed antibiotics in the previous 12-months had poorer knowledge of the use of antibiotics to treat colds/flu and COVID-19. Furthermore, the proportion of individuals stating that they do not know whether antibiotics can be used to treat ear infections increased by 21% from pre- to post-pandemic. This proportion also increased by 9% for the question relating to effectiveness of antibiotics at treating colds/flu.

One of the key findings from this comparison over time are the increases in uncertainty observed across all knowledge statements post-pandemic. The longevity of improvements in knowledge following public campaigns is unclear [12]; therefore the increased levels of uncertainty seen following the COVID-19 pandemic may result from a lack of focus on this area during the pandemic when the majority of public messaging focused on infection prevention behaviours, such as hand hygiene and social distancing. This uncertainty highlights potential opportunities to educate the public by revitalising previous campaigns with refreshed, direct messaging, as is done for successful marketing campaigns [29], which may improve knowledge and attitudes and increase the public’s confidence in their knowledge of this area. Future public campaigns should draw upon lessons learnt from the COVID-19 pandemic, to present consistent messaging [21, 28] that recognises the role of human behaviours [21, 29], provided from a trusted source [21, 28–30] to underpin AMS and emphasise the roles that individuals can take in tackling AMR.

In addition to changes in knowledge and attitudes, while trust in HCPs has previously been reported as being high [30], the present study shows this peaked during the pandemic, possibly due to the critical nature of the situation [31, 32] and the increased levels of respect for and appreciation of HCPs which was evident across the UK through public behaviours such as ‘Clap for Carers’ [33, 34]. The absence of public campaigns combined with associations observed between trust, compliance with health advice and treatment, and patient satisfaction, may have contributed towards the increased knowledge of appropriate antibiotic use seen during the pandemic, as well as the reduction in the proportion of respondents who stated they were likely to request antibiotics from their GPs [35].

Information regarding antibiotic efficacy has been shown to reduce patient expectation for antibiotics [36, 37]. There is also some evidence to suggest that higher trust in HCPs may enhance the effect [36] suggesting HCPs are well placed to educate patients and to provide them with skills needed to self-care for self-limiting infections. Therefore, future interventions may wish to educate HCPs on how to further improve trust with their patients, such as through their communication skills, demonstrating their level of professional competence and showing empathy with their patients [38]. Increasing HCPs awareness of supporting resources may also increase the likelihood that they would provide advice to patients, although lack of time has also frequently been cited as a barrier to this [5, 10].

The present study highlighted several groups which had consistently poorer knowledge regarding AMR and antibiotic use, including those from lower social grades (C2DE), respondents from ethnic minority backgrounds, those educated to less than degree level, and younger age groups. This concurs with previous survey findings [15, 30]. The present study also shows consistently lower levels of trust in HCPs in respondents from ethnic minority backgrounds compared to white respondents. It is important to understand the causes of poorer knowledge and lower levels of trust within these groups as many have been shown to experience higher risk of some bacterial infections, such as *Staphylococcus aureus* and *Mycobacterium tuberculosis*, and resistant infections, as well as higher rates of antibiotic use [5, 39, 40].

### Strengths and limitations

This work furthers our understanding of how public knowledge and attitudes have changed over the COVID-19 pandemic. It also provides an overview of the current landscape at the start of the second UK NAP, highlighting areas for improvement that will help achieve targets set out within national policy. Furthermore, by exploring the patient element of the patient-prescriber dynamic, our work can inform public campaigns and training on positive interactions between HCPs and patients to improve public knowledge of AMR and appropriate antibiotic use.

One strength of this work is the sampling methodologies used with random quota sampling (used from pre-pandemic to pandemic-Y2) and weighting of questionnaire responses increasing the likelihood of the questionnaire responses being representative, and therefore generalisable to the wider English population. However, while the demographics of the sample may have been representative it cannot be determined whether non-response bias occurred or whether respondents differed systematically from the wider population resulting in volunteer bias [41]. Furthermore, the sample size obtained for ethnic minority backgrounds within the pre-pandemic, pandemic-Y1 and pandemic-Y2 questionnaires was not sufficient to perform sub-group using individual ethnic backgrounds. As a result, these respondents were grouped into a single ethnic minority variable. Whilst this methodology is not ideal, it is important to highlight any differences between demographics. Future public survey work will look to ensure robust samples within ethic minority groups, as has been done for the post-pandemic survey, to allow for differences to be determined within individual ethnic minority backgrounds. Another limitation relates to the change in questionnaire structure across the years with some questions not included within the 2024 questionnaire.

Finally, the change in data collection methods from interviewer-administered to a self-completion survey may have resulted in a change in the kinds of participants that took part in the survey. For example, the online access panel approach used in 2024 excludes people who are not on the internet, while this population would have been included in the face-to-face and telephone surveys of 2020, 2021 and 2022. The change in participant profile can be adjusted to some extent, most notably by weighting the samples to be nationally representative of the population in England. However, this cannot correct for all differences in the kinds of participants who took part. It may also have impacted on the proportion of respondents selecting don’t know as individuals may feel more comfortable admitting a lack of knowledge on a top when completing a self-administered questionnaire.

## Conclusion

This paper shows that despite increases during the COVID-19 pandemic, knowledge and attitudes regarding AMR and antibiotic use has returned to pre-pandemic levels. This increase in knowledge was very short-lived given the considerable public awareness-raising on viruses. A large increase in uncertainty regarding the specific infections that antibiotics can be used to treat was evident post-pandemic. Furthermore, differences in knowledge were seen among populations with factors associated with health inequalities. This provides information on potential areas of and populations to focus public engagement work on in order to achieve targets and commitments set out in the 2024-29 UK NAP. Further work is needed to determine the impact increased knowledge and positive attitudes towards AMR and antibiotic use have on an individual’s subsequent antibiotic usage behaviours and on overall prescribing rates within the UK.

This paper also demonstrates the high levels of public trust in HCPs, with respondents who trusted their GPs advice on whether an antibiotic was needed being less likely to challenge their GPs decision regarding their need for antibiotics. Trust has declined following the COVID-19 pandemic; therefore, interventions may wish to support HCPs to build trust with their patients and consider how care pathways can promote trust. They could also look to improve HCP awareness of available resources that facilitate meaningful discussions with patients on antibiotic use. Finally, there is a clear ongoing need for funded, targeted public education campaigns.

## Supplementary Information

Supplementary Material 1.

## Data Availability

The datasets used and/or analysed during the current study are available from the corresponding author on reasonable request.

## Abbreviations

AMR: Antimicrobial resistance
AMS: Antimicrobial stewardship
CAPI: Computer-aided personal interviews
CATI: Computer-aided telephone interviews
CAWI: Computer assisted web interviewing
COVID-19: Coronavirus disease that emerged in 2019
ESPAUR: English surveillance programme for antimicrobial utilisation and resistance
GP: General practitioner
HCP: Healthcare professionals
IMD: Index of multiple deprivation
NAP: National action plan
RCGP: Royal college of general practitioners
TARGET: Treat Antibiotics responsibly; guidance, education and tools
UK: United Kingdom
