# Supplementary material for: Public knowledge and attitudes towards antibiotic use across England – pre- and post-pandemic
Source: BMC Public Health. 2025 Nov 21;25:4078. doi: 10.1186/s12889-025-25233-3 (PMC12639946; doi:10.1186/s12889-025-25233-3)
Supplement: Supplementary file 1 — Supplementary Material 1. [file 12889_2025_25233_MOESM1_ESM.docx]

## Supplementary Material 1. Additional information on sampling techniques and calculation of respondent demographic groups

## **Sampling**

### *For 2020 face-to-face sampling*

Two-stage random location sampling was used. The initial sampling frame is a bespoke amalgamation of Output Areas (used for output from the Census in Great Britain) which are regrouped into Primary Sampling Units (PSUs) taking account of their ACORN (A Classification of Residential Neighbourhood) characteristics. The use of ACORN ensures all types of area are fully represented and respondent selection is not determined by interviewers, helping to eliminate any possible bias in the sample caused by interviewing people all with the same background. A total of 170-180 of these PSUs are then randomly selected with probability of selection proportional to size. At the second stage, from each PSU typically two adjacent output areas are randomly selected, each area includes about 125 addresses. Interviewers were given age, gender, household tenure, and working status quotas of participants. Households were visited throughout the day and week; partially completed interviews were excluded (if participants terminated the interview). Interviewers go door-to-door and invite people who are at home and are over 15 years old to participate (the interview does not proceed if the respondent falls within a filled quota). Only one individual is interviewed per household. Interviewers do not revisit non-responding households. One interview is completed on average for every 3-4 doors knocked.

*For 2021 and 2022 telephone sampling*

We source two types of data for the telephone omnibus. Targeted data which includes a name and Random Digital Dial data (RDD) which is just a phone number. Targeted data is sourced from feeds from 18 sources of opted in and publicly available data: including electoral roll, competitions, warranties, and online forms. Other demographic information is overlaid from 11 subsequent feeds, to understand types of property, levels deprivation levels etc. Records are selected from across the UK (including or excluding Northern Ireland), whilst the records are selected at random, they are tied to geographical and demographical information to ensure it is representative of each Government Office Region in the UK based on latest census information. All records with a number are made available for selection unless they have been called within a window of 6 months. As some individuals would typically be excluded from this methodology due to never completing forms (for example those new to the UK), we provide an additional feed of RDD data, this data is based upon a 'start point', for example 01926 (Leamington) or 07931 (EE), numbers are then at random added to these stubs to create a full number. To understand the geography of the landlines we cross reference the area code 01926 Leamington and Warwick exchange with the stub e.g., 315 Lillington and the randomly create the last digitals starting with 01926315000, then 001, 002 etc. (many numbers will not connect as they are not assigned). The process is similar for mobile numbers where we randomly generate 6 additional characters, we then validate these numbers for connectivity which provides a last known geography. With RDD we have no intelligence on the demographic of the individual being called as we only have a number, therefore we must extract many more numbers than are requested to make the data geographically representative and screening questions have to be applied to capture other demographics when capturing people's views.

### *Demographics*

Social grade of the household is determined by the occupation of the chief income earner. Social grade AB comprises high or intermediate managerial, administrative, or professional workers; C1 Supervisory, clerical, and junior managerial, administrative or professional workers; C2 skilled manual workers; D semi/ unskilled manual workers; E State pensioners, casual or lowest grade workers, unemployed with state benefits only.

A respondent’s highest level of educational attainment was classified according to general certificate of secondary education (GCSE); further education (A-level or equivalent); higher education (degree or equivalents).

Respondents who did not self-identify as white (which accounts for ~15% of the population in England) were and responses compared from white and non-white respondents were compared. The authors would like to address that dichotomising ethnicity into only two variables is crude and assumes heterogeneity amongst all non-white groups. Future surveys must oversample across all non-white groups to allow adequate power to more appropriately analyse ethnicity to reflect the diversity of the UK.

Supplementary Material 2. Unweighted respondent demographics for 2020, 2021, 2022 and 2024 on the left, weighted respondent demographics on the right

|  | | 2020 (Pre-pandemic) | | | | 2021 (Pandemic Y-1) | | | | 2022 (Pandemic-Y2 | | | | 2024 (Post-pandemic) | | | |
| --- | --- | --- | --- | --- | --- | --- | --- | --- | --- | --- | --- | --- | --- | --- | --- | --- | --- |
|  |  | Unweighted | | Weighted | | Unweighted | | Weighted | | Unweighted | | Weighted | | Unweighted | | Weighted | |
|  |  | n | % | n | % | n | % | n | % | n | % | n | % | n | % | n | % |
| Gender | Female | 1026 | 50.7 | 1035 | 50.9 | 931 | 55.5 | 852 | 50.8 | 820 | 49.3 | 840 | 50.5 | 1603 | 53.0 | 1551 | 51.3 |
|  | Male | 996 | 49.3 | 997 | 49.1 | 736 | 43.9 | 815 | 48.6 | 828 | 49.8 | 808 | 48.6 | 1383 | 45.7 | 1429 | 47.3 |
| Age | 15-24 | 406 | 20.1 | 291 | 14.3 | 161 | 9.6 | 238 | 14.2 | 190 | 11.4 | 225 | 13.5 | 39 | 1.3 | 425 | 14.1 |
|  | 25-34 | 266 | 13.2 | 340 | 16.7 | 238 | 14.2 | 276 | 16.5 | 266 | 16.0 | 282 | 17.0 | 565 | 18.7 | 519 | 17.2 |
|  | 35-44 | 267 | 13.2 | 310 | 15.3 | 257 | 15.3 | 256 | 15.3 | 213 | 12.8 | 260 | 15.6 | 565 | 18.7 | 458 | 15.1 |
|  | 45-54 | 290 | 14.3 | 338 | 16.6 | 307 | 18.3 | 277 | 16.5 | 255 | 15.3 | 274 | 16.5 | 539 | 17.8 | 473 | 15.6 |
|  | 55-64 | 312 | 15.4 | 296 | 14.6 | 263 | 15.7 | 247 | 14.7 | 297 | 17.9 | 246 | 14.8 | 498 | 16.5 | 462 | 15.3 |
|  | 65+ | 481 | 23.8 | 458 | 22.5 | 446 | 26.6 | 378 | 22.6 | 441 | 26.5 | 376 | 22.6 | 467 | 15.4 | 686 | 22.7 |
| Social Grade | ABC1 | 1277 | 63.2 | 1135 | 55.9 | 999 | 59.6 | 848 | 50.6 | 1006 | 60.5 | 838 | 50.4 | 1998 | 66.1 | 1751 | 57.9 |
|  | C2DE | 745 | 36.8 | 897 | 44.1 | 569 | 33.9 | 750 | 44.7 | 516 | 31.0 | 726 | 43.7 | 1026 | 33.9 | 1273 | 42.1 |
| Education | Below degree level | 1172 | 58.0 | 1183 | 58.2 | 1044 | 62.3 | 1056 | 63.0 | 884 | 53.2 | 911 | 54.8 | 1364 | 45.1 | 2027 | 67.0 |
|  | Degree | 635 | 31.4 | 661 | 32.5 | 609 | 36.3 | 597 | 35.6 | 752 | 45.2 | 723 | 43.5 | 1660 | 54.9 | 997 | 33.0 |
| Ethnicity | White | 1488 | 73.6 | 1727 | 8.5 | 1473 | 87.9 | 1445 | 86.2 | 1430 | 86.0 | 1393 | 83.8 | 1778 | 58.8 | 2506 | 82.9 |
|  | All ethnic minorities | 521 | 25.8 | 290 | 14.3 | 194 | 11.6 | 222 | 13.2 | 213 | 12.8 | 247 | 14.9 | 1223 | 40.4 | 497 | 16.4 |
|  | Black | 119 | 5.9 | 55 | 2.7 | 56 | 3.3 | 66 | 3.9 | 67 | 4.0 | 79 | 4.8 | NR | NR | NR | NR |
|  | Asian | 320 | 15.8 | 193 | 9.5 | 78 | 4.7 | 84 | 5.0 | 85 | 5.1 | 98 | 5.9 | NR | NR | NR | NR |
|  | Mixed | 47 | 2.3 | 24 | 1.2 | 40 | 2.4 | 49 | 2.9 | 41 | 2.5 | 46 | 2.8 | NR | NR | NR | NR |
|  | Other | 35 | 1.7 | 18 | 0.9 | 20 | 1.2 | 23 | 1.4 | 20 | 1.2 | 23 | 1.4 | NR | NR | NR | NR |
| Pre-pandemic, unweight n = 2,022, weighted n = 2032 ; Pandemic-Y1, unweighted and weighted n = 1,676; Pandemic-Y2, unweighted and weighted n = 1,663; Post-pandemic, unweighted and weighted n = 3,024 | | | | | | | | | | | | | | | | | |

Supplementary Material 3. Responses to knowledge and attitudes questions in 2020, 2021, 2022 and 2024

|  | | 2020 (Pre-pandemic) | | 2021 (Pandemic Y-1) | | 2022 (Pandemic-Y2 | | 2024 (Post-pandemic) | |
| --- | --- | --- | --- | --- | --- | --- | --- | --- | --- |
|  |  | n | % | n | % | n | % | n | % |
| There's nothing I can personally do to prevent antibiotics from becoming less effective at treating infections | True (Incorrect) | 785 | 38.6 | 522 | 31.1 | 568 | 34.2 | 1289 | 25.9 |
|  | False (Correct) | 1159 | 57.0 | 1041 | 62.1 | 948 | 57.0 | 2295 | 46.1 |
|  | Unsure | 88 | 4.3 | 113 | 6.7 | 147 | 8.8 | 1391 | 28.0 |
| If there is any doubt about whether an infection needs to be treated with antibiotics it's better to take them just in case | True (Incorrect) | 375 | 18.5 | 625 | 37.3 | 619 | 37.2 | NC | NC |
|  | False (Correct) | 1268 | 62.4 | 966 | 57.6 | 933 | 56.1 | NC | NC |
|  | Unsure | 67 | 3.3 | 85 | 5.1 | 111 | 6.7 | NC | NC |
| Antibiotics always speed up my recovery, no matter what the infection is | True (Incorrect) | 777 | 38.2 | 494 | 29.5 | 479 | 28.8 | 1181 | 23.7 |
|  | False (Correct) | 1181 | 58.1 | 1092 | 65.2 | 1083 | 65.1 | 2779 | 55.8 |
|  | Unsure | 74 | 3.6 | 89 | 5.3 | 101 | 6.1 | 1016 | 20.4 |
| There is nothing society can do to prevent antibiotics from becoming less effective at treating infections | True (Incorrect) | 630 | 31.0 | 429 | 25.6 | 488 | 29.3 | NC | NC |
|  | False (Correct) | 1303 | 64.1 | 1120 | 66.8 | 1024 | 61.6 | NC | NC |
|  | Unsure | 99 | 4.9 | 127 | 7.6 | 151 | 9.1 | NC | NC |
| Antibiotics will always work when you really need them to | True (Incorrect) | 794 | 39.1 | 618 | 36.9 | 579 | 34.8 | NC | NC |
|  | False (Correct) | 1173 | 57.7 | 985 | 58.8 | 1002 | 60.3 | NC | NC |
|  | Unsure | 66 | 3.2 | 73 | 4.4 | 82 | 4.9 | NC | NC |
| Antibiotics work for the majority of ear infections | True (Incorrect) | 1383 | 68.1 | 1340 | 80.0 | 1290 | 77.6 | 2865 | 57.6 |
|  | False (Correct) | 431 | 21.2 | 172 | 10.3 | 184 | 11.1 | 500 | 10.0 |
|  | Unsure | 218 | 10.7 | 164 | 9.8 | 189 | 11.4 | 1611 | 32.4 |
| Antibiotics work for the majority of urine infections | True (Correct) | 1537 | 75.6 | 1421 | 84.8 | 1379 | 82.9 | 3218 | 64.7 |
|  | False (Incorrect) | 301 | 14.8 | 96 | 5.7 | 108 | 6.5 | 283 | 5.7 |
|  | Unsure | 194 | 9.5 | 159 | 9.5 | 176 | 10.6 | 1474 | 29.6 |
| Antibiotics work for the symptoms of COVID-19 | True (Incorrect) | NC | NC | 242 | 14.4 | 262 | 15.8 | 543 | 10.9 |
|  | False (Correct) | NC | NC | 1193 | 71.2 | 1162 | 69.9 | 3141 | 63.1 |
|  | Unsure | NC | NC | 241 | 14.4 | 238 | 14.3 | 1291 | 25.9 |
| Antibiotics work for the majority cold or flu viruses | True (Incorrect) | 569 | 28.0 | 466 | 27.8 | 395 | 23.8 | 828 | 16.6 |
|  | False (Correct) | 1406 | 69.2 | 1125 | 67.1 | 1170 | 70.4 | 3529 | 70.9 |
|  | Unsure | 57 | 2.8 | 84 | 5.0 | 98 | 5.9 | 619 | 12.4 |
| I trust my GP's advice on whether or not I need antibiotics | Agree | 1768 | 87.0 | 1530 | 91.3 | 1494 | 89.8 | 4299 | 86.4 |
|  | Neither agree nor disagree | 90 | 4.4 | 58 | 3.5 | 77 | 4.6 | 439 | 8.8 |
|  | Disagree | 147 | 7.2 | 84 | 5.0 | 77 | 4.6 | 206 | 4.1 |
|  | Unsure | 27 | 1.3 | 5 | 0.3 | 15 | 0.9 | 33 | 0.7 |
| I trust my nurse's advice on whether or not I need antibiotics | Agree | 1536 | 75.6 | 1405 | 83.8 | 1341 | 80.6 | 3704 | 74.4 |
|  | Neither agree nor disagree | 204 | 10.0 | 116 | 6.9 | 148 | 8.9 | 875 | 17.6 |
|  | Disagree | 239 | 11.8 | 147 | 8.8 | 150 | 9.0 | 317 | 6.4 |
|  | Unsure | 53 | 2.6 | 7 | 0.4 | 24 | 1.4 | 80 | 1.6 |
| I trust my pharmacist's advice on whether or not I need antibiotics | Agree | 1433 | 70.5 | 1279 | 76.3 | 1277 | 76.8 | 3597 | 72.3 |
|  | Neither agree nor disagree | 245 | 12.1 | 161 | 9.6 | 163 | 9.8 | 878 | 17.6 |
|  | Disagree | 301 | 14.8 | 215 | 12.8 | 195 | 11.7 | 381 | 7.7 |
|  | Unsure | 52 | 2.6 | 21 | 1.3 | 29 | 1.7 | 119 | 2.4 |
| I would be pleased if the GP said I didn’t need antibiotics for an infection | Agree | 1711 | 84.2 | 1392 | 83.1 | 1317 | 79.2 | 3246 | 65.2 |
|  | Neither agree nor disagree | NC | NC | NC | NC | NC | NC | 1140 | 22.9 |
|  | Disagree | 261 | 12.8 | 200 | 11.9 | 218 | 13.1 | 449 | 9.0 |
|  | Unsure | 60 | 3.0 | 84 | 5.0 | 128 | 7.7 | 140 | 2.8 |
| How likely would you be to ask the GP to prescribe antibiotics, even if they have said they are not needed to treat your infection | Likely | 427 | 21.0 | 315 | 18.8 | 356 | 21.4 | 1266 | 25.4 |
|  | Not likely | 1573 | 77.4 | 1337 | 79.8 | 1265 | 76.1 | 3366 | 67.6 |
|  | Unsure | 31 | 1.5 | 24 | 1.4 | 42 | 2.5 | 343 | 6.9 |
| Pre-pandemic, n = 2,022; Pandemic-Y1, n = 1,676; Pandemic-Y2, n = 1,663; Post-pandemic, n = 4,976 | | | | | | | | | |

Supplementary Material 4. Responses to knowledge and attitudes questions in 2020, 2021, 2022 and 2024 for each social grade

|  |  | 2020 (Pre-pandemic) | | 2021 (Pandemic Y-1) | | 2022 (Pandemic-Y2 | | 2024 (Post-pandemic) | |
| --- | --- | --- | --- | --- | --- | --- | --- | --- | --- |
|  |  | n | % | n | % | n | % | n | % |
| There's nothing I can personally do to prevent antibiotics from becoming less effective at treating infections | | | | | | | | | |
| ABC1 | TRUE | 358 | 32 | 229 | 27 | 242 | 29 | 431 | 25 |
|  | FALSE | 740* | 65* | 575* | 68* | 543* | 65* | 844 | 48 |
|  | Unsure | 38 | 3 | 45 | 5 | 54 | 6 | 476 | 27 |
| C2DE | TRUE | 427* | 48* | 271* | 36* | 291* | 40* | 352 | 28 |
|  | FALSE | 419 | 47 | 424 | 57 | 361 | 50 | 551 | 43 |
|  | Unsure | 51 | 6 | 55 | 7 | 74* | 10* | 370 | 29 |
| If there is any doubt about whether an infection needs to be treated with antibiotics it's better to take them just in case | | | | | | | | | |
| ABC1 | TRUE | 319 | 28 | 287 | 34 | 275 | 33 | NC | NC |
|  | FALSE | 785* | 69* | 526* | 62* | 510* | 61* | NC | NC |
|  | Unsure | 31 | 3 | 35 | 4 | 53 | 6 | NC | NC |
| C2DE | TRUE | 379* | 42* | 313* | 42* | 305* | 42* | NC | NC |
|  | FALSE | 483 | 54 | 396 | 53 | 379 | 52 | NC | NC |
|  | Unsure | 36 | 4 | 41 | 5 | 43 | 6 | NC | NC |
| Antibiotics always speed up my recovery, no matter what the infection is | | | | | | | | | |
| ABC1 | TRUE | 327 | 29 | 206 | 24 | 192 | 23 | 375 | 21 |
|  | FALSE | 769* | 68* | 607* | 72* | 613* | 73* | 1024* | 58* |
|  | Unsure | 38 | 3 | 36 | 4 | 33 | 4 | 352 | 20 |
| C2DE | TRUE | 449* | 50* | 268* | 36* | 254* | 35* | 342* | 27* |
|  | FALSE | 412 | 46 | 435 | 58 | 419 | 58 | 665 | 52 |
|  | Unsure | 36 | 4 | 46 | 6 | 53* | 7* | 265 | 21 |
| There is nothing society can do to prevent antibiotics from becoming less effective at treating infections | | | | | | | | | |
| ABC1 | TRUE | 257 | 23 | 165 | 19 | 193 | 23 | NC | NC |
|  | FALSE | 842* | 74* | 632* | 75* | 589* | 70* | NC | NC |
|  | Unsure | 36 | 3 | 51 | 6 | 56 | 7 | NC | NC |
| C2DE | TRUE | 373* | 42* | 243* | 32* | 267* | 37* | NC | NC |
|  | FALSE | 461 | 51 | 446 | 59 | 383 | 53 | NC | NC |
|  | Unsure | 63 | 7 | 61 | 8 | 76* | 11* | NC | NC |
| Antibiotics will always work when you really need them to | | | | | | | | | |
| ABC1 | TRUE | 371 | 33 | 272 | 32 | 243 | 29 | NC | NC |
|  | FALSE | 734* | 65* | 545* | 64* | 561* | 67* | NC | NC |
|  | Unsure | 29 | 3 | 31 | 4 | 35 | 4 | NC | NC |
| C2DE | TRUE | 423* | 47* | 323* | 43* | 305* | 42* | NC | NC |
|  | FALSE | 438 | 49 | 391 | 52 | 388 | 53 | NC | NC |
|  | Unsure | 37 | 4 | 36 | 5 | 33 | 5 | NC | NC |
| Antibiotics work for the majority of ear infections | | | | | | | | | |
| ABC1 | TRUE | 759 | 67 | 679 | 80 | 641 | 77 | 969 | 55 |
|  | FALSE | 239 | 21 | 91 | 11 | 110* | 13* | 181 | 10 |
|  | Unsure | 137 | 12 | 78 | 9 | 82 | 10 | 601 | 34 |
| C2DE | TRUE | 624 | 55 | 606 | 81 | 572 | 79 | 772* | 61* |
|  | FALSE | 193 | 17 | 70 | 9 | 63 | 9 | 122 | 10 |
|  | Unsure | 81 | 7 | 74 | 10 | 92 | 13 | 378 | 30 |
| Antibiotics work for the majority of urine infections | | | | | | | | | |
| ABC1 | TRUE | 855 | 75 | 736 | 87 | 702 | 84 | 1112 | 63 |
|  | FALSE | 168 | 15 | 47 | 6 | 55 | 7 | 100 | 6 |
|  | Unsure | 112 | 10 | 65 | 8 | 81 | 10 | 539 | 31 |
| C2DE | TRUE | 681 | 76 | 624 | 83 | 601 | 83 | 844 | 66 |
|  | FALSE | 133 | 15 | 41 | 5 | 47 | 6 | 72 | 6 |
|  | Unsure | 82 | 9 | 84* | 11* | 78 | 11 | 356 | 28 |
| Antibiotics don’t work for COVID-19 | | | | | | | | | |
| ABC1 | TRUE | NC | NC | 96 | 11 | 103 | 12 | 178 | 10 |
|  | FALSE | NC | NC | 654* | 77* | 642* | 77* | 1126 | 64 |
|  | Unsure | NC | NC | 98 | 12 | 93 | 11 | 447 | 26 |
| C2DE | TRUE | NC | NC | 139* | 19* | 143* | 20* | 152 | 12 |
|  | FALSE | NC | NC | 485 | 65 | 463 | 64 | 783 | 62 |
|  | Unsure | NC | NC | 126* | 17* | 120* | 17* | 338 | 27 |
| Antibiotics work for the majority cold or flu viruses | | | | | | | | | |
| ABC1 | TRUE | 238 | 21 | 200 | 24 | 153 | 18 | 265 | 15 |
|  | FALSE | 881* | 78* | 610* | 72* | 647* | 77* | 1263 | 72 |
|  | Unsure | 16 | 1 | 38 | 4 | 38 | 5 | 223 | 13 |
| C2DE | TRUE | 331* | 37* | 243* | 32* | 213* | 29* | 238 | 19 |
|  | FALSE | 526 | 59 | 466 | 62 | 467 | 64 | 881 | 69 |
|  | Unsure | 40 | 4 | 41 | 5 | 47 | 6 | 153 | 12 |
| How likely would you be to ask the GP to prescribe antibiotics, even if they have said they are not needed to treat your infection | | | | | | | | | |
| ABC1 | Likely | 190 | 17 | 145 | 17 | 148 | 18 | 413 | 24 |
|  | Not likely | 938* | 83* | 693 | 82 | 676* | 81* | 1222* | 70* |
|  | Unsure | 8 | 1 | 10 | 1 | 14 | 2 | 116 | 7 |
| C2DE | Likely | 236* | 26* | 154 | 20 | 185* | 25* | 357 | 28 |
|  | Not likely | 636 | 71 | 586 | 78 | 523 | 72 | 824 | 65 |
|  | Unsure | 25 | 3 | 11 | 1 | 18 | 3 | 92 | 7 |

Supplementary Material 5. Responses to knowledge and attitudes questions in 2020, 2021, 2022 and 2024 for each education level

|  |  | 2020 (Pre-pandemic) | | 2021 (Pandemic Y-1) | | 2022 (Pandemic-Y2 | | 2024 (Post-pandemic) | |
| --- | --- | --- | --- | --- | --- | --- | --- | --- | --- |
|  |  | n | % | n | % | n | % | n | % |
| There's nothing I can personally do to prevent antibiotics from becoming less effective at treating infections | | | | | | | | | |
| Less than degree | TRUE | 537 | 45 | 403* | 38* | 371* | 41* | 542 | 27 |
|  | FALSE | 592 | 50 | 572 | 54 | 448 | 49 | 861 | 42 |
|  | Unsure | 53 | 4 | 81* | 8* | 93* | 10* | 624* | 31* |
| Degree | TRUE | 177 | 27 | 111 | 19 | 188 | 26 | 241 | 24 |
|  | FALSE | 464* | 70* | 459* | 77* | 487* | 67* | 534* | 54* |
|  | Unsure | 20 | 3 | 27 | 5 | 48 | 7 | 222 | 22 |
| If there is any doubt about whether an infection needs to be treated with antibiotics it's better to take them just in case | | | | | | | | | |
| Less than degree | TRUE | 443* | 37* | 428* | 41* | 382* | 42* | NC | NC |
|  | FALSE | 693 | 59 | 570 | 54 | 468 | 51 | NC | NC |
|  | Unsure | 47 | 4 | 58 | 6 | 61 | 7 | NC | NC |
| Degree | TRUE | 167 | 25 | 186 | 31 | 222 | 31 | NC | NC |
|  | FALSE | 482* | 73* | 389* | 65* | 454* | 63* | NC | NC |
|  | Unsure | 12 | 2 | 22 | 4 | 47 | 6 | NC | NC |
| Antibiotics always speed up my recovery, no matter what the infection is | | | | | | | | | |
| Less than degree | TRUE | 498* | 42* | 364* | 34* | 320* | 35* | 504 | 25 |
|  | FALSE | 629 | 53 | 621 | 59 | 532 | 58 | 1085 | 54 |
|  | Unsure | 55 | 5 | 71* | 7* | 59 | 6 | 439 | 22 |
| Degree | TRUE | 178 | 27 | 119 | 20 | 148 | 21 | 214 | 21 |
|  | FALSE | 472* | 71* | 462* | 77* | 540* | 75* | 604* | 61* |
|  | Unsure | 11 | 2 | 16 | 3 | 35 | 5 | 179 | 18 |
| There is nothing society can do to prevent antibiotics from becoming less effective at treating infections | | | | | | | | | |
| Less than degree | TRUE | 439* | 37* | 324* | 31* | 336* | 37* | NC | NC |
|  | FALSE | 665 | 56 | 641 | 61 | 485 | 53 | NC | NC |
|  | Unsure | 77 | 7 | 91* | 9* | 90* | 10* | NC | NC |
| Degree | TRUE | 122 | 18 | 96 | 16 | 146 | 20 | NC | NC |
|  | FALSE | 527* | 80* | 470* | 79* | 527* | 73* | NC | NC |
|  | Unsure | 13 | 2 | 31 | 5 | 50 | 7 | NC | NC |
| Antibiotics will always work when you really need them to | | | | | | | | | |
| Less than degree | TRUE | 514* | 43* | 443* | 42* | 373* | 41* | NC | NC |
|  | FALSE | 627 | 53 | 563 | 53 | 487 | 53 | NC | NC |
|  | Unsure | 42 | 4 | 51 | 5 | 51 | 6 | NC | NC |
| Degree | TRUE | 195 | 30 | 164 | 28 | 193 | 27 | NC | NC |
|  | FALSE | 449* | 68* | 415* | 70* | 502* | 69* | NC | NC |
|  | Unsure | 17 | 3 | 18 | 3 | 28 | 4 | NC | NC |
| Antibiotics work for the majority of ear infections | | | | | | | | | |
| Less than degree | TRUE | 824 | 70 | 863* | 82* | 719 | 79 | 1156 | 57 |
|  | FALSE | 234 | 20 | 85 | 8 | 86 | 9 | 191 | 9 |
|  | Unsure | 124 | 10 | 108 | 10 | 106 | 12 | 680 | 34 |
| Degree | TRUE | 444 | 67 | 461 | 77 | 555 | 77 | 586 | 59 |
|  | FALSE | 145 | 22 | 88* | 15* | 95* | 13* | 113 | 11 |
|  | Unsure | 72 | 11 | 49 | 8 | 73 | 10 | 299 | 30 |
| Antibiotics work for the majority of urine infections | | | | | | | | | |
| Less than degree | TRUE | 912 | 77 | 907 | 86 | 766 | 84 | 1316 | 65 |
|  | FALSE | 158 | 13 | 46 | 4 | 54 | 6 | 103 | 5 |
|  | Unsure | 112 | 9 | 103 | 10 | 91 | 10 | 608 | 30 |
| Degree | TRUE | 487 | 74 | 496 | 83 | 592 | 82 | 640 | 64 |
|  | FALSE | 106 | 16 | 49* | 8* | 53 | 7 | 69 | 7 |
|  | Unsure | 68 | 10 | 51 | 9 | 78 | 11 | 287 | 29 |
| Antibiotics don’t work for COVID-19 | | | | | | | | | |
| Less than degree | TRUE | NC | NC | 181* | 17* | 179* | 20* | 194 | 10 |
|  | FALSE | NC | NC | 704 | 67 | 586 | 64 | 1246 | 61 |
|  | Unsure | NC | NC | 171* | 16* | 147* | 16* | 587* | 29* |
| Degree | TRUE | NC | NC | 57 | 10 | 79 | 11 | 136* | 14* |
|  | FALSE | NC | NC | 481* | 81* | 566* | 78* | 664* | 67* |
|  | Unsure | NC | NC | 59 | 10 | 79 | 11 | 198 | 20 |
| Antibiotics work for the majority cold or flu viruses | | | | | | | | | |
| Less than degree | TRUE | 369* | 31* | 332* | 31* | 268* | 29* | 325 | 16 |
|  | FALSE | 772 | 65 | 670 | 63 | 581 | 64 | 1436 | 71 |
|  | Unsure | 41 | 3 | 54 | 5 | 62* | 7* | 266 | 13 |
| Degree | TRUE | 129 | 19 | 124 | 21 | 119 | 16 | 179 | 18 |
|  | FALSE | 524* | 79* | 448* | 75* | 574* | 79* | 709 | 71 |
|  | Unsure | 8 | 1 | 25 | 4 | 31 | 4 | 110 | 11 |

Supplementary Material 6. Responses to knowledge and attitudes questions in 2020, 2021, 2022 and 2024 by ethnicity

|  |  | 2020 (Pre-pandemic) | | 2021 (Pandemic-Y1) | | 2022 (Pandemic-Y2) | | 2024 (Post-pandemic) | |
| --- | --- | --- | --- | --- | --- | --- | --- | --- | --- |
|  |  | n | % | n | % | n | % | n | % |
| There's nothing I can personally do to prevent antibiotics from becoming less effective at treating infections | | | | | | | | | |
| White | TRUE | 633 | 37 | 439 | 30 | 454 | 33 | 615 | 25 |
|  | FALSE | 1025* | 59* | 908 | 63 | 820* | 59* | 1188* | 47* |
|  | Unsure | 69 | 4 | 98 | 7 | 119 | 9 | 703 | 28 |
| Ethnic Minorities | TRUE | 146* | 50* | 81 | 37 | 104* | 42* | 163* | 33* |
|  | FALSE | 125 | 43 | 128 | 58 | 120 | 49 | 198 | 40 |
|  | Unsure | 20 | 7 | 13 | 6 | 22 | 9 | 136 | 27 |
| If there is any doubt about whether an infection needs to be treated with antibiotics it's better to take them just in case | | | | | | | | | |
| White | TRUE | 559 | 32 | 526 | 36 | 513 | 37 | NC | NC |
|  | FALSE | 1117* | 65* | 844 | 58 | 785 | 56 | NC | NC |
|  | Unsure | 51 | 3 | 75 | 5 | 95 | 7 | NC | NC |
| Ethnic Minorities | TRUE | 133* | 46* | 95 | 43 | 104 | 42 | NC | NC |
|  | FALSE | 141 | 49 | 119 | 54 | 128 | 52 | NC | NC |
|  | Unsure | 16* | 6* | 8 | 4 | 14 | 6 | NC | NC |
| Antibiotics always speed up my recovery, no matter what the infection is | | | | | | | | | |
| White | TRUE | 609 | 35 | 418 | 29 | 377 | 27 | 537 | 21 |
|  | FALSE | 1055* | 61* | 947 | 66 | 937* | 67* | 1464* | 58* |
|  | Unsure | 62 | 4 | 80 | 6 | 79 | 6 | 506 | 20 |
| Ethnic Minorities | TRUE | 159* | 55* | 76 | 34 | 98* | 40* | 172* | 35* |
|  | FALSE | 119 | 41 | 140 | 63 | 130 | 52 | 217 | 44 |
|  | Unsure | 12 | 4 | 6 | 3 | 19 | 8 | 107 | 22 |
| There is nothing society can do to prevent antibiotics from becoming less effective at treating infections | | | | | | | | | |
| White | TRUE | 482 | 28 | 369 | 26 | 389 | 28 | NC | NC |
|  | FALSE | 1164* | 67* | 971 | 67 | 880* | 63* | NC | NC |
|  | Unsure | 81 | 5 | 105 | 7 | 124 | 9 | NC | NC |
| Ethnic Minorities | TRUE | 143* | 49* | 59 | 26 | 86 | 35 | NC | NC |
|  | FALSE | 129 | 44 | 145 | 65 | 135 | 55 | NC | NC |
|  | Unsure | 18 | 6 | 19 | 8 | 26 | 11 | NC | NC |
| Antibiotics will always work when you really need them to | | | | | | | | | |
| White | TRUE | 621 | 36 | 530 | 37 | 484 | 35 | NC | NC |
|  | FALSE | 1054* | 61* | 851 | 59 | 839 | 60 | NC | NC |
|  | Unsure | 51 | 3 | 63 | 4 | 70 | 5 | NC | NC |
| Ethnic Minorities | TRUE | 165* | 57* | 85 | 38 | 92 | 37 | NC | NC |
|  | FALSE | 111 | 38 | 130 | 58 | 144 | 58 | NC | NC |
|  | Unsure | 14 | 5 | 8 | 4 | 11 | 5 | NC | NC |
| Antibiotics work for the majority of ear infections | | | | | | | | | |
| White | TRUE | 1171 | 68 | 1163 | 80 | 1085 | 78 | 1418 | 57 |
|  | FALSE | 365 | 21 | 138 | 10 | 159 | 11 | 265* | 11* |
|  | Unsure | 190 | 11 | 144 | 10 | 149 | 11 | 823 | 33 |
| Ethnic Minorities | TRUE | 198 | 68 | 172 | 78 | 187 | 76 | 315* | 63* |
|  | FALSE | 64 | 22 | 33* | 15* | 24 | 10 | 36 | 7 |
|  | Unsure | 28 | 10 | 17 | 8 | 36 | 15 | 146 | 29 |
| Antibiotics work for the majority of urine infections | | | | | | | | | |
| White | TRUE | 1313 | 76 | 1235 | 85 | 1165 | 84 | 1611 | 64 |
|  | FALSE | 246 | 14 | 75 | 5 | 84 | 6 | 132 | 5 |
|  | Unsure | 167 | 10 | 135 | 9 | 145 | 10 | 763* | 30* |
| Ethnic Minorities | TRUE | 211 | 73 | 179 | 81 | 195 | 79 | 336 | 68 |
|  | FALSE | 51 | 18 | 21* | 9* | 24 | 10 | 39* | 8* |
|  | Unsure | 28 | 10 | 22 | 10 | 27 | 11 | 122 | 24 |
| Antibiotics don’t work for COVID-19 | | | | | | | | | |
| White | TRUE | NC | NC | 195 | 13 | 196 | 14 | 243 | 10 |
|  | FALSE | NC | NC | 1034 | 72 | 999* | 72* | 1631* | 65* |
|  | Unsure | NC | NC | 216 | 15 | 198 | 14 | 632 | 25 |
| Ethnic Minorities | TRUE | NC | NC | 46* | 21* | 63* | 26* | 86* | 17* |
|  | FALSE | NC | NC | 155 | 70 | 148 | 60 | 271 | 55 |
|  | Unsure | NC | NC | 21 | 9 | 36 | 14 | 140 | 28 |
| Antibiotics work for the majority cold or flu viruses | | | | | | | | | |
| White | TRUE | 401 | 23 | 362 | 25 | 274 | 20 | 337 | 13 |
|  | FALSE | 1281* | 74* | 1013* | 70* | 1039* | 75* | 1880* | 75* |
|  | Unsure | 44 | 3 | 70 | 5 | 80 | 6 | 289 | 12 |
| Ethnic Minorities | TRUE | 159* | 55* | 104* | 47* | 116* | 47* | 161* | 32* |
|  | FALSE | 118 | 41 | 103 | 47 | 116 | 47 | 256 | 52 |
|  | Unsure | 12 | 4 | 15 | 7 | 15 | 6 | 79* | 16* |
| I trust my GP's advice as to whether I need antibiotics or not | | | | | | | | | |
| White | Agree | 1530* | 89* | 1333* | 92* | 1262 | 91 | 2201* | 88* |
|  | Don't agree | 197 | 11 | 113 | 8 | 131 | 9 | 306 | 12 |
| Ethnic Minorities | Agree | 225 | 78 | 188 | 85 | 215 | 87 | 398 | 80 |
|  | Don't agree | 65* | 22* | 34* | 15* | 32 | 13 | 98 | 20 |
| I trust my nurses's advice as to whether I need antibiotics or not | | | | | | | | | |
| White | Agree | 1342* | 78* | 1224* | 85* | 1142* | 82* | 1908* | 76* |
|  | Don't agree | 385 | 22 | 221 | 15 | 251 | 18 | 599 | 24 |
| Ethnic Minorities | Agree | 180 | 62 | 172 | 77 | 182 | 74 | 331 | 67 |
|  | Don't agree | 110* | 38* | 50* | 23* | 64* | 26* | 167 | 33 |
| I trust my pharmacist's advice as to whether I need antibiotics or not | | | | | | | | | |
| White | Agree | 1234* | 71* | 1116 | 77 | 1084 | 78 | 1833* | 73* |
|  | Don't agree | 492 | 29 | 329 | 23 | 309 | 22 | 673 | 27 |
| Ethnic Minorities | Agree | 187 | 65 | 156 | 70 | 179 | 72 | 338 | 68 |
|  | Don't agree | 103* | 35* | 66 | 30 | 68 | 28 | 159 | 32 |
| How likely would you be to ask the GP to prescribe antibiotics, even if they have said they are not needed to treat your infection | | | | | | | | | |
| White | Likely | 318 | 18 | 238 | 16 | 268 | 19 | 556 | 22 |
|  | Not likely | 1383* | 80* | 1188* | 82* | 1090* | 78* | 1779* | 71* |
|  | Unsure | 26 | 2 | 19 | 1 | 35 | 3 | 171 | 7 |
| Ethnic Minorities | Likely | 107* | 37* | 75* | 34* | 86* | 35* | 205* | 41* |
|  | Not likely | 178 | 61 | 142 | 64 | 155 | 63 | 257 | 52 |
|  | Unsure | 5 | 2 | 5 | 2 | 6 | 2 | 35 | 7 |

Supplementary Material 7. Responses to knowledge and attitudes questions in 2020, 2021, 2022 and 2024 for each age group

|  |  | 2020 (Pre-pandemic) | | 2021 (Pandemic Y-1) | | 2022 (Pandemic-Y2 | | 2024 (Post-pandemic) | |
| --- | --- | --- | --- | --- | --- | --- | --- | --- | --- |
|  |  | n | % | n | % | n | % | n | % |
| There's nothing I can personally do to prevent antibiotics from becoming less effective at treating infections | | | | | | | | | |
| 15-24 a | TRUE | 127 | 44 | 86bcd | 36bcd | 92de | 41de | 119e | 28e |
|  | FALSE | 156 | 54 | 145f | 61f | 114 | 51 | 173 | 41 |
| 25-34 b | TRUE | 112 | 33 | 65 | 23 | 94 | 33 | 170ce | 33ce |
|  | FALSE | 212f | 63f | 197ef | 71ef | 166f | 59f | 227 | 44 |
| 35-44 c | TRUE | 103 | 33 | 54 | 21 | 85 | 33 | 102 | 22 |
|  | FALSE | 196af | 63af | 187aef | 73aef | 158f | 61f | 236ad | 51ad |
| 45-54 d | TRUE | 108 | 32 | 71 | 26 | 79 | 29 | 129e | 27e |
|  | FALSE | 220aef | 65aef | 192f | 69f | 171af | 62af | 196 | 41 |
| 55-64 e | TRUE | 122 | 41 | 74c | 30c | 76 | 31 | 76 | 17 |
|  | FALSE | 161 | 54 | 153f | 62f | 153af | 62af | 250abdf | 54abdf |
| Over 65 f | TRUE | 213 | 47 | 171bcde | 45bcde | 142d | 38d | 188e | 27e |
|  | FALSE | 213 | 47 | 164 | 43 | 187 | 50 | 314 | 46 |
| If there is any doubt about whether an infection needs to be treated with antibiotics it's better to take them just in case | | | | | | | | | |
| 15-24 a | TRUE | 111e | 38e | 89 | 37 | 94 | 42 | NC | NC |
|  | FALSE | 170 | 59 | 141f | 59f | 125 | 55 | NC | NC |
| 25-34 b | TRUE | 138e | 41e | 86 | 31 | 106 | 38 | NC | NC |
|  | FALSE | 195 | 57 | 180ef | 65ef | 157 | 56 | NC | NC |
| 35-44 c | TRUE | 100 | 32 | 76 | 30 | 92 | 36 | NC | NC |
|  | FALSE | 201 | 65 | 173ef | 68ef | 145 | 56 | NC | NC |
| 45-54 d | TRUE | 110 | 33 | 107c | 39c | 102 | 37 | NC | NC |
|  | FALSE | 222 | 66 | 160f | 58f | 153 | 56 | NC | NC |
| 55-64 e | TRUE | 86 | 29 | 101bc | 41bc | 84 | 34 | NC | NC |
|  | FALSE | 194 | 66 | 133 | 54 | 145 | 59 | NC | NC |
| Over 65 f | TRUE | 152 | 33 | 165bc | 44bc | 141 | 38 | NC | NC |
|  | FALSE | 287 | 63 | 176 | 47 | 209 | 55 | NC | NC |
| Antibiotics always speed up my recovery, no matter what the infection is | | | | | | | | | |
| 15-24 a | TRUE | 117 | 40 | 67 | 28 | 78d | 34d | 115f | 27f |
|  | FALSE | 168f | 58f | 166f | 70f | 139 | 62 | 233 | 55 |
| 25-34 b | TRUE | 137 | 40 | 62 | 23 | 81 | 29 | 163def | 31def |
|  | FALSE | 194 | 57 | 209ef | 76ef | 192f | 68f | 273 | 53 |
| 35-44 c | TRUE | 104 | 33 | 50 | 20 | 70 | 27 | 112 | 24 |
|  | FALSE | 196f | 63f | 196def | 77def | 170 | 65 | 240 | 52 |
| 45-54 d | TRUE | 113 | 33 | 74 | 27 | 63 | 23 | 110 | 23 |
|  | FALSE | 220f | 65f | 190f | 69f | 187f | 68f | 253 | 53 |
| 55-64 e | TRUE | 100 | 34 | 77bc | 31bc | 62 | 25 | 92 | 20 |
|  | FALSE | 182f | 62f | 152f | 62f | 173f | 71f | 259 | 56 |
| Over 65 f | TRUE | 206cde | 45cde | 164abcde | 43abcde | 125de | 33de | 125 | 18 |
|  | FALSE | 222 | 49 | 176 | 47 | 222 | 59 | 432bcd | 63bcd |
| There is nothing society can do to prevent antibiotics from becoming less effective at treating infections | | | | | | | | | |
| 15-24 a | TRUE | 106 | 31 | 74 | 31 | 93bcde | 42bcde | NC | NC |
|  | FALSE | 170 | 58 | 149f | 63f | 112 | 50 | NC | NC |
| 25-34 b | TRUE | 106 | 31 | 65 | 23 | 78 | 28 | NC | NC |
|  | FALSE | 219 | 65 | 195f | 71f | 179af | 64af | NC | NC |
| 35-44 c | TRUE | 84 | 27 | 45 | 18 | 62 | 24 | NC | NC |
|  | FALSE | 210af | 68af | 194af | 76af | 183af | 70af | NC | NC |
| 45-54 d | TRUE | 94 | 28 | 62 | 22 | 69 | 25 | NC | NC |
|  | FALSE | 235af | 69af | 203af | 73af | 178af | af | NC | NC |
| 55-64 e | TRUE | 73 | 25 | 54 | 22 | 58 | 24 | NC | NC |
|  | FALSE | 208af | 70af | 181af | 73af | 169af | 69af | NC | NC |
| Over 65 f | TRUE | 167cde | 36cde | 128bcde | 34bcde | 129cde | 34cde | NC | NC |
|  | FALSE | 261 | 57 | 194 | 51 | 203 | 54 | NC | NC |
| Antibiotics will always work when you really need them to | | | | | | | | | |
| 15-24 a | TRUE | 113 | 39 | 65 | 27 | 83 | 37 | NC | NC |
|  | FALSE | 172 | 59 | 164def | 69def | 132 | 59 | NC | NC |
| 25-34 b | TRUE | 137 | 40 | 86 | 31 | 96 | 34 | NC | NC |
|  | FALSE | 194 | 57 | 182ef | 66ef | 175f | 62f | NC | NC |
| 35-44 c | TRUE | 127 | 41 | 75 | 29 | 83 | 32 | NC | NC |
|  | FALSE | 166 | 54 | 176def | 69def | 162 | 63 | NC | NC |
| 45-54 d | TRUE | 109 | 32 | 100 | 36 | 86 | 31 | NC | NC |
|  | FALSE | 222cf | 66cf | 163f | 59f | 177f | 65f | NC | NC |
| 55-64 e | TRUE | 106 | 36 | 102abc | 41abc | 83 | 34 | NC | NC |
|  | FALSE | 182f | 61f | 138f | 56f | 152f | 62f | NC | NC |
| Over 65 f | TRUE | 202de | 44de | 187abcde | 50abcde | 148d | 39d | NC | NC |
|  | FALSE | 238 | 52 | 160 | 42 | 203 | 54 | NC | NC |
| Antibiotics work for the majority of ear infections | | | | | | | | | |
| 15-24 a | TRUE | 211c | 73c | 193 | 81 | 191def | 85def | 254 | 60 |
|  | FALSE | 59 | 20 | 31f | 13f | 12 | 5 | 42 | 10 |
| 25-34 b | TRUE | 236 | 69 | 223 | 81 | 232ef | 82ef | 371cdef | 71cdef |
|  | FALSE | 71 | 21 | 37f | 13f | 27 | 10 | 37 | 7 |
| 35-44 c | TRUE | 198 | 64 | 208 | 81 | 209e | 80e | 240 | 52 |
|  | FALSE | 78 | 25 | 31f | 12f | 28 | 11 | 79abdef | 17abdef |
| 45-54 d | TRUE | 233 | 69 | 219 | 79 | 208 | 76 | 267 | 56 |
|  | FALSE | 77 | 23 | 26 | 9 | 38a | 14a | 47 | 10 |
| 55-64 e | TRUE | 200 | 68 | 205 | 83 | 177 | 72 | 247 | 53 |
|  | FALSE | 64 | 22 | 20 | 8 | 37a | 15a | 39 | 8 |
| Over 65 f | TRUE | 305 | 67 | 289 | 77 | 274 | 73 | 363 | 53 |
|  | FALSE | 83 | 18 | 27 | 7 | 43a | 11a | 60 | 9 |
| Antibiotics work for the majority of urine infections | | | | | | | | | |
| 15-24 a | TRUE | 222 | 76 | 196 | 82 | 183 | 81 | 247 | 58 |
|  | FALSE | 48f | 17f | 22ef | 9ef | 18 | 8 | 34f | 8f |
| 25-34 b | TRUE | 253 | 75 | 235 | 85 | 236 | 84 | 368ac | 71ac |
|  | FALSE | 54 | 16 | 24ef | 9ef | 19 | 7 | 39f | 7f |
| 35-44 c | TRUE | 223 | 72 | 215 | 84 | 217 | 84 | 272 | 59 |
|  | FALSE | 51 | 17 | 17f | 7f | 19 | 7 | 31f | 7f |
| 45-54 d | TRUE | 260 | 77 | 235 | 85 | 225 | 82 | 301 | 64 |
|  | FALSE | 54 | 16 | 14 | 5 | 18 | 7 | 25 | 5 |
| 55-64 e | TRUE | 225 | 76 | 218 | 88 | 206 | 84 | 305 | 66 |
|  | FALSE | 42 | 14 | 8 | 3 | 16 | 7 | 22 | 5 |
| Over 65 f | TRUE | 354 | 77 | 320 | 85 | 312 | 8 | 463c | 68c |
|  | FALSE | 51 | 11 | 10 | 3 | 18 | 5 | 21 | 3 |
| Antibiotics don’t work for COVID-19 | | | | | | | | | |
| 15-24 a | TRUE | NC | NC | 47 | 20 | 57bcdef | 25bcdef | 61def | 14def |
|  | FALSE | NC | NC | 163f | 68f | 140 | 62 | 241 | 57 |
| 25-34 b | TRUE | NC | NC | 40 | 14 | 43 | 15 | 133acdef | 26acdef |
|  | FALSE | NC | NC | 204f | 74f | 200 | 71 | 266 | 51 |
| 35-44 c | TRUE | NC | NC | 32 | 12 | 35 | 13 | 50ef | 11ef |
|  | FALSE | NC | NC | 206aef | 80aef | 197af | 76af | 292b | 64b |
| 45-54 d | TRUE | NC | NC | 28 | 10 | 31 | 11 | 30 | 6 |
|  | FALSE | NC | NC | 218af | 79af | 199af | 73af | 320ab | 68ab |
| 55-64 e | TRUE | NC | NC | 30 | 12 | 37 | 15 | 23 | 5 |
|  | FALSE | NC | NC | 178f | 72f | 186af | 76af | 329ab | 71ab |
| Over 65 f | TRUE | NC | NC | 67 | 18 | 59 | 16 | 33 | 5 |
|  | FALSE | NC | NC | 222 | 59 | 240 | 64 | 461ab | 67ab |
| Antibiotics work for the majority cold or flu viruses | | | | | | | | | |
| 15-24 a | TRUE | 127ef | 44ef | 129 | 54 | 113bcdef | 50bcdef | 142cdef | 33cdef |
|  | FALSE | 157 | 54 | 93 | 39 | 98 | 44 | 224 | 53 |
| 25-34 b | TRUE | 107e | 31e | 85 | 31 | 85cdef | 30cdef | 181cdef | 35cdef |
|  | FALSE | 221a | 65a | 176a | 64a | 178a | 63a | 261 | 50 |
| 35-44 c | TRUE | 83e | 27e | 49 | 19 | 48 | 19 | 77def | 17def |
|  | FALSE | 219a | 71a | 198abf | 77abf | 196ab | 75ab | 307ab | 67ab |
| 45-54 d | TRUE | 82 | 24 | 41 | 15 | 50 | 18 | 42f | 9f |
|  | FALSE | 253ab | 75ab | 226abf | 82abf | 204ab | 75ab | 364abc | 77abc |
| 55-64 e | TRUE | 56 | 19 | 45 | 18 | 35 | 14 | 30 | 6 |
|  | FALSE | 230ab | 78ab | 190abf | 77abf | 203abd | 83abd | 386abcd | 84abcd |
| Over 65 f | TRUE | 113 | 25 | 116 | 31 | 64 | 17 | 32 | 5 |
|  | FALSE | 326a | 71a | 240a | 63a | 290ab | 77ab | 603abcd | 88abcd |
| I trust my GP's advice as to whether I need antibiotics or not | | | | | | | | | |
| 15-24 a | Agree | 254 | 87 | 210 | 88 | 198 | 88 | 349 | 82 |
|  | Don't agree | 37 | 13 | 28e | 12e | 27 | 12 | 77 | 18 |
| 25-34 b | Agree | 289 | 85 | 237 | 86 | 243 | 86 | 425 | 82 |
|  | Don't agree | 51 | 15 | 39def | 14def | 39f | 14f | 93 | 18 |
| 35-44 c | Agree | 259 | 83 | 234 | 91 | 230 | 89 | 380 | 83 |
|  | Don't agree | 52 | 17 | 22 | 9 | 30 | 11 | 78 | 17 |
| 45-54 d | Agree | 296 | 87 | 257b | 93b | 250 | 91 | 406 | 86 |
|  | Don't agree | 42 | 13 | 20 | 7 | 24 | 9 | 68 | 14 |
| 55-64 e | Agree | 263 | 89 | 236ab | 96ab | 225 | 92 | 416abc | 90abc |
|  | Don't agree | 32 | 1 | 11 | 4 | 20 | 8 | 47 | 10 |
| Over 65 f | Agree | 407 | 89 | 353b | 93b | 348b | 93b | 637abcd | 93abcd |
|  | Don't agree | 50 | 11 | 25 | 7 | 28 | 7 | 49 | 7 |
| I trust my nurse's advice as to whether I need antibiotics or not | | | | | | | | | |
| 15-24 a | Agree | 237f | 81f | 210 | 88 | 176 | 78 | 328 | 77 |
|  | Don't agree | 54 | 19 | 28 | 12 | 49 | 22 | 97 | 23 |
| 25-34 b | Agree | 252 | 74 | 229 | 83 | 226 | 80 | 374 | 72 |
|  | Don't agree | 88 | 26 | 48 | 17 | 56 | 20 | 145 | 28 |
| 35-44 c | Agree | 230 | 74 | 214 | 84 | 220f | 85f | 344 | 75 |
|  | Don't agree | 81 | 26 | 41 | 16 | 39 | 15 | 114 | 25 |
| 45-54 d | Agree | 255 | 76 | 234 | 84 | 222 | 81 | 362 | 76 |
|  | Don't agree | 83 | 24 | 43 | 16 | 52 | 19 | 111 | 24 |
| 55-64 e | Agree | 226 | 76 | 208 | 84 | 205 | 84 | 337 | 73 |
|  | Don't agree | 70 | 24 | 38 | 16 | 40 | 16 | 125 | 27 |
| Over 65 f | Agree | 336 | 73 | 308 | 81 | 292 | 78 | 506 | 74 |
|  | Don't agree | 121a | 27a | 70 | 19 | 84c | 22c | 180 | 26 |
| I trust my pharmacist's advice as to whether I need antibiotics or not | | | | | | | | | |
| 15-24 a | Agree | 209 | 72 | 197bf | 83bf | 180 | 80 | 314 | 74 |
|  | Don't agree | 82 | 28 | 41 | 17 | 45 | 20 | 112 | 26 |
| 25-34 b | Agree | 235 | 69 | 196 | 71 | 209 | 74 | 380 | 73 |
|  | Don't agree | 104 | 31 | 81a | 29a | 73 | 26 | 138 | 27 |
| 35-44 c | Agree | 211 | 68 | 202 | 79 | 198 | 76 | 317 | 69 |
|  | Don't agree | 99 | 32 | 54 | 21 | 61 | 24 | 30 | 31 |
| 45-54 d | Agree | 248 | 73 | 218 | 79 | 207 | 76 | 343 | 72 |
|  | Don't agree | 90 | 27 | 59 | 21 | 67 | 24 | 131 | 28 |
| 55-64 e | Agree | 215 | 73 | 189 | 76 | 189 | 77 | 332 | 72 |
|  | Don't agree | 80 | 27 | 58 | 24 | 57 | 23 | 130 | 28 |
| Over 65 f | Agree | 315 | 69 | 278 | 74 | 294 | 78 | 501 | 73 |
|  | Don't agree | 142 | 31 | 100a | 26a | 82 | 22 | 186 | 27 |
| I would be pleased if the GP said I didn't need antibiotics for an infection | | | | | | | | | |
| 15-24 a | TRUE | 231 | 79 | 184 | 77 | 180 | 80 | 244 | 57 |
|  | FALSE | 50cde | 17cde | 45def | 19def | 33 | 15 | 62def | 15def |
| 25-34 b | TRUE | 271 | 80 | 226 | 82 | 206 | 73 | 309 | 60 |
|  | FALSE | 61cde | 18cde | 33 | 12 | 53def | 19def | 78cdef | cdef |
| 35-44 c | TRUE | 264 | 85 | 209 | 81 | 192 | 74 | 277 | 60 |
|  | FALSE | 31 | 10 | 33 | 13 | 42e | 16e | 39f | 8f |
| 45-54 d | TRUE | 300ab | 89ab | 239a | 86a | 229bc | 83bc | 330abc | 70abc |
|  | FALSE | 35 | 10 | 26 | 9 | 28 | 10 | 30 | 6 |
| 55-64 e | TRUE | 260ab | 88ab | 215a | 87a | 204bc | 83bc | 314a | 68a |
|  | FALSE | 27 | 9 | 24 | 10 | 23 | 9 | 36 | 8 |
| Over 65 f | TRUE | 386 | 84 | 316 | 84 | 306bc | 81bc | 500abc | 73abc |
|  | FALSE | 57 | 12 | 39 | 10 | 40 | 11 | 27 | 4 |
| How likely would you be to ask the GP to prescribe antibiotics, even if they have said they are not needed to treat your infection | | | | | | | | | |
| 15-24 a | Likely | 59 | 20 | 60ef | 25ef | 64def | 29def | 171def | 40def |
|  | Not likely | 227 | 78 | 174 | 73 | 156 | 69 | 238 | 56 |
| 25-34 b | Likely | 87e | 26e | 58 | 21 | 69e | 24e | 205def | 40def |
|  | Not likely | 250 | 73 | 214 | 77 | 209 | 74 | 281 | 54 |
| 35-44 c | Likely | 75e | 24e | 55d | 22d | 59 | 23 | 157def | 34def |
|  | Not likely | 229 | 74 | 199 | 78 | 195 | 75 | 272 | 59 |
| 45-54 d | Likely | 72 | 21 | 41 | 15 | 51 | 19 | 104ef | 22ef |
|  | Not likely | 264 | 78 | 232a | 84a | 218a | 79a | 315ab | 67ab |
| 55-64 e | Likely | 49 | 16 | 39 | 16 | 41 | 17 | 63 | 14 |
|  | Not likely | 242bc | 82bc | 207a | 84a | 195a | 79a | 368abcd | 80abcd |
| Over 65 f | Likely | 86 | 19 | 60 | 16 | 71 | 19 | 69 | 10 |
|  | Not likely | 362 | 79 | 307 | 81 | 292 | 78 | 571abcd | 83abcd |

Supplementary Material 8. Responses to knowledge questions regarding the use of antibiotics to treat specific infections in 2020, 2021, 2022 and 2024 by gender

|  |  | 2020 (Pre-pandemic) | | 2021 (Pandemic-Y1) | | 2022 (Pandemic-Y2) | | 2024 (Post-pandemic) | |
| --- | --- | --- | --- | --- | --- | --- | --- | --- | --- |
|  |  | n | % | n | % | n | % | n | % |
| Antibiotics work for the majority of ear infections | | | | | | | | | |
| Females | TRUE | 735* | 71* | 693 | 81 | 682* | 81* | 967* | 62* |
|  | FALSE | 204 | 20 | 87 | 10 | 84 | 10 | 153 | 10 |
|  | Unsure | 97 | 9 | 72 | 8 | 75 | 9 | 432 | 28 |
| Males | TRUE | 648 | 65 | 640 | 79 | 596 | 74 | 747 | 52 |
|  | FALSE | 228 | 23 | 85 | 10 | 100 | 12 | 146 | 10 |
|  | Unsure | 121 | 12 | 90 | 11 | 111* | 14* | 536* | 38* |
| Antibiotics work for the majority of urine infections | | | | | | | | | |
| Females | TRUE | 806* | 78* | 745* | 87* | 723* | 86* | 1115* | 72* |
|  | FALSE | 144 | 14 | 54 | 6 | 56 | 7 | 82 | 5 |
|  | Unsure | 86 | 8 | 52 | 6 | 60 | 7 | 354 | 23 |
| Males | TRUE | 732 | 73 | 671 | 82 | 645 | 80 | 811 | 57 |
|  | FALSE | 157 | 16 | 42 | 5 | 51 | 6 | 85 | 6 |
|  | Unsure | 109 | 11 | 103* | 13* | 113* | 14* | 533* | 37* |
| Antibiotics don’t work for COVID-19 | | | | | | | | | |
| Females | TRUE | NC | NC | 109 | 13 | 108 | 13 | 197* | 14* |
|  | FALSE | NC | NC | 613 | 72 | 609* | 73* | 834 | 58 |
|  | Unsure | NC | NC | 130 | 15 | 123 | 15 | 398 | 28 |
| Males | TRUE | NC | NC | 131 | 16 | 152* | 19* | 128 | 8 |
|  | FALSE | NC | NC | 575 | 71 | 544 | 67 | 1048* | 68* |
|  | Unsure | NC | NC | 109 | 13 | 112 | 14 | 376 | 24 |
| Antibiotics work for the majority cold or flu viruses | | | | | | | | | |
| Females | TRUE | 252 | 24 | 195 | 23 | 149 | 18 | 308* | 22* |
|  | FALSE | 757* | 73* | 620* | 73* | 655* | 78* | 914 | 64 |
|  | Unsure | 26 | 2 | 38 | 4 | 36 | 4 | 207* | 14* |
| Males | TRUE | 316* | 32* | 267* | 33* | 245* | 30* | 187 | 12 |
|  | FALSE | 649 | 65 | 502 | 62 | 505 | 63 | 1199* | 77* |
|  | Unsure | 31 | 3 | 46 | 6 | 57* | 7* | 166 | 11 |
